# Fractionation of Golgi, Endoplasmic Reticulum, and Plasma Membrane from Cultured Cells in a Preformed Continuous Iodixanol Gradient

**DOI:** 10.1100/tsw.2002.286

**Published:** 2002-05-22

**Authors:** John Graham

**Affiliations:** School of Biomolecular Sciences, Liverpool John Moores University, USA

## Abstract

A continuous iodixanol gradient within the range 0-30% (w/v) iodixanol can resolve the major membrane compartments of the endoplasmic reticulum, Golgi membranes, and plasma membrane from a postnuclear supernatant prepared from a cultured cell homogenate. The precise density range of the gradient and the centrifugation conditions (100,000-200,000 *g* for 2-16 h) vary with the type of cell and the requirements of the separation. The strategy is widely used to study the processing of proteins within cells.

**METHOD TYPE:** extraction, isolation, purification and separation

**SUB METHOD TYPE:** centrifugation

## INTRODUCTION

The protocol is adapted from the methods published by Yang et al.[1] and Zhang et al.[2] who used rather similar gradients for quite different purposes. Yang et al.[1] used a 0–26% linear iodixanol gradient to study the localization of UBC6 ubiquitin-containing protein in COS-7 cells. By using the gradient to separate ER and Golgi, they established that the transmembrane domain of a carboxyl-terminal–anchored protein predisposes it to locate to the ER, while modulation of this domain resulted in retargeting of the protein to the Golgi.

Zhang et al.[2] used a 1–20% linear iodixanol gradient also to separate the ER and Golgi from transfected CHO and human embryonic kidney 293 cells. The authors showed that the full-length presenilins (PS1 and PS2) were located in the ER while N- and C-terminal fragments were distributed to the Golgi membranes.

These linear preformed gradients may be top- or bottom-loaded with the sample, although in both of the published methods top-loading was used. The vesicle fraction applied to the gradient may be as a low-speed supernatant[1] or as a resuspended vesicle pellet[2].

## MATERIALS AND EQUIPMENT

OptiPrep™
Homogenization medium (HM): 0.25 M sucrose, 1 mM EDTA 10 mM Hepes-NaOH, pH 7.4 (see Note 1).
Diluent: 0.25 M sucrose, 6 mM EDTA, 60 mM Hepes-NaOH, pH 7.4.
Working Solution (WS) of 50% (w/v) iodixanol ($\rho$ = 1.272 g/ml): 5 vol of OptiPrep™ + 1 vol of Diluent (see Note 2).

Ball-bearing homogenizer or other suitable device (see Note 3)
Refrigerated low- or high-speed centrifuge with swinging-bucket or fixed-angle rotor (2–10 ml tubes)
Ultracentrifuge with swinging-bucket rotor (approx. 200,000 *g*) with tube volumes of approx. 14 ml (see Note 4). Fixed-angle rotor is also required for optional step 4.
Gradient Master or two-chamber gradient maker.
Gradient unloader; for tube puncture or upward displacement with a dense medium.

## METHOD

Carry out all operations at 0–4°C.

1. Wash the cells twice in phosphate-buffered saline to remove the culture medium, and then once in the homogenization medium before resuspending in this medium (see Note 5).
2. Suspend the cells in a small volume of HM (0.5–5.0 ml) and disrupt them using the homogenizer of choice (see Note 6).
3. Centrifuge the homogenate at 1,000–3,000 *g* for 10 min. The pellet may be resuspended in homogenization medium; the centrifugation repeated and the two supernatants combined, if necessary (see Note 7).
4. **Optional step.** Centrifuge the supernatant(s) at 100,000 *g* for 40 min and then resuspend the pellet in 1–2 ml of HM.
5. Prepare a 25% (w/v) iodixanol solution by mixing equal volumes of HM and WS.
6. Prepare 12- to 13-ml gradients in tubes for the swinging-bucket rotor from equal volumes of HM and the 25% iodixanol solution using a two-chamber gradient maker or a Gradient Master (see Note 8).
7. Layer the vesicle suspension on top of the gradient and centrifuge at 200,000 *g* for 2–3 h (see Note 9).
8. Collect the gradient in 0.5 fractions either by tube puncture or upward displacement.

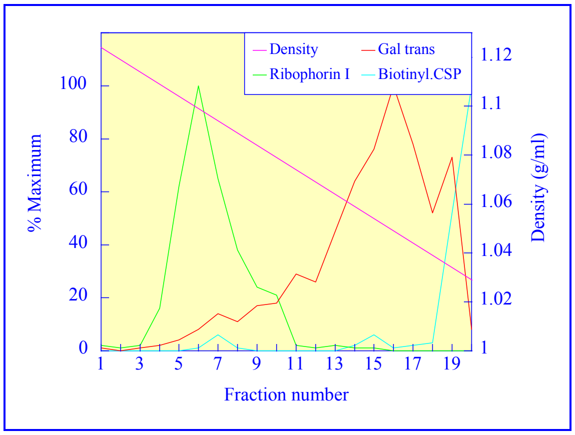

**FIGURE 1**. Distribution of Golgi, ER, and plasma membrane markers from CHO cells in a 0–26% iodixanol gradient (115 min at 200,000 $g_{av}$. Gal trans = galactosyl transferase; Biotinyl.CSP = biotinylated cell surface proteins. Figure adapted from Ref. 1 with kind permission of the American Society for Biochemistry and Molecular Biology and the authors.

## ANALYSIS

A typical distribution of plasma membrane (biotinylated cell surface proteins), Golgi (galactosyl transferase), and ER (ribophorin I) markers is shown Fig. 1. Plasma membrane bands at the very top of the gradient, the Golgi is broadly banded in the top third, and the ER in the bottom third of the gradient (see Note 10).

## NOTES

1. With cultured cells the homogenization medium often has to be tailored to the cell type. Sometimes an alternative buffer (10 mM triethanolamine-acetic acid) achieves a better cell disruption. For more information see Ref. 3.
2. Strategies for preparing working solutions for mammalian tissues and cells are given in Ref. 4. Protease inhibitors may be included in any or all of the media at the operator's discretion
3. The ball-bearing homogenizer (cell-cracker) is certainly the preferred device, but passage through a fine (25-gauge) syringe needle or Dounce (tight-fitting Wheaton Type A pestle) homogenization are acceptable alternatives[3].
4. Other swinging-bucket rotors or even vertical rotors may be used. Larger volume swinging-bucket rotors will require longer centrifugation times but vertical rotors will need shorter times.
5. Washing the cells with saline may be carried out at room temperature rather than at 4°C if preferred. Yang et al.[1] used a modified homogenization buffer containing 0.21 M sucrose, 0.75 mM KCl, 19 mM NaCl, 1 mM EDTA to wash the cells prior to homogenization.
6. In all cases, however, the optimal number of passages through the ball-bearing homogenizer (4–10 is common) or syringe needle (10–12 is common), or the number of passes of the pestle (10–20 is common) of the Dounce homogenizer need to be determined empirically for each specific cell type. For more information about cell homogenization see Ref. 3.

7. The advantage of using a low-speed supernatant for the gradient input is that the procedure is quicker and that vesicles in the supernatant are not further exposed to the shearing forces that are required to resuspend a 100,000-*g* pellet. On the other hand if it is important to remove any soluble cytosolic proteins then preparing a 100,000-*g* pellet may be a useful step.
8. If neither of these devices is available, then a continuous gradient can be prepared by diffusion of a discontinuous gradient[5]. The actual density range of the gradient can be customized to the operator's requirements. If the main interest is the Golgi and lower density fractions then a 0–15% iodixanol gradient may be more useful.
9. There may be instances where it is beneficial to reduce the centrifugation time or RCF, if some of the vesicles are to be separated on the basis of size (i.e., conditions where the vesicles do not reach their banding density).
10. Some other examples of the use of iodixanol gradients for fractionating these membrane compartments are given in Table 1.

**TABLE 1**
**Separation of ER, Golgi, and PM using Preformed Iodixanol Gradient (Selected Publications)**

| Gradient | Centrifugation | Event/Separation | Ref. |
|---|---|---|---|
| Disc 2.5-30% | 180,000 *g*/2.5 h | Processing of β-amyloid precursor protein in ER and Golgi | [6] |
| Cont 9-30% | 52,000 *g*/90 min | Colocalization of Cholera toxin-$A_1$ peptide to PDI-containing ER fraction | [7] |
| Cont 10-30% | 45,000 *g*/16 h | Very high resolution of ER and Golgi – localization of UDPases | [8] |
| Cont 10-30% | 200,000 *g*/3 h | Processing of β-amyloid precursor protein | [9] |
| Cont 8-22% | 100,000 *g*/2 h | Residence of cytochrome b(5) in ER | [10] |
| Disc 2.5-30% | 180,000 *g*/2.5 h | ER, *cis*-Golgi localization of HTLV-1 | [11] |
| Cont 0-26% | 196,000 *g*/2.5 h | Localization of fusion proteins of β-gal. and HIV-1 transmembrane protein cytoplasmic tail | [12] |

## ACKNOWLEDGEMENTS

The author and TheScientificWorld wish to thank Dr Allan Weissman, Laboratory of Immune Cell Biology, National Cancer Institute, Bethesda, MD 20892 and Dr Edward Koo, Department of Neurosciences 0691, University of California at San Diego, La Jolla, CA 92093 for their cooperation in the preparation of this text in the preparation of this Protocol Article and Axis-Shield PoC, AS, Oslo, Norway for their kind permission to adapt OptiPrep™ Application Sheet S18 and Table 35 from the Axis-Shield Catalogue 2002.
